# Early eGFR decline in DAPA-CKD: causal structure, subgrouping, and the risk of post-randomization bias

**DOI:** 10.1093/ckj/sfag149

**Published:** 2026-05-07

**Authors:** Ali Zeynettin

**Affiliations:** Department of İnternal Medicine, Izmir City Hospital, Izmir, Turkiye

To the Editor,

The specific target of this critique is the post hoc Dapagliflozin and Prevention of Adverse Outcomes in Chronic Kidney Disease (DAPA-CKD) analysis in which acute estimated glomerular filtration rate (eGFR) change from baseline to Week 2 was categorized as >10% decline, >0% to 10% decline, or increase in eGFR, with subsequent eGFR trajectories and clinical outcomes evaluated across these postrandomization strata [1]. The overall renal benefit of dapagliflozin in DAPA-CKD is well established [2]. However, these *post hoc* analyses have been interpreted as clinically reassuring, suggesting that an early eGFR dip does not attenuate long-term renal benefit and supporting continuation of therapy despite an acute haemodynamic dip. Nevertheless, the analytical handling of early eGFR decline warrants evaluation within an explicit causal framework.

Early eGFR change is a postrandomization variable directly influenced by treatment assignment while simultaneously reflecting baseline renal haemodynamic reserve, which is itself associated with long-term renal prognosis. Conditioning on such an intermediate variable introduces a risk of collider bias [3]. Classifying patients as ‘dip’ and ‘nondip’ may therefore distort the treatment–outcome relationship by opening noncausal pathways. Although randomization ensures baseline balance, subgroup analyses based on postrandomization variables inherently weaken causal interpretation. Importantly, these issues arise even in fully randomized settings, underscoring that randomization alone does not protect against bias introduced by postrandomization conditioning.

A simple causal thought experiment or proof-of-concept simulation can illustrate how conditioning on a postrandomization variable may induce collider bias. Such an illustration is not intended to reproduce DAPA-CKD data, but rather to provide a conceptual illustration of how apparent heterogeneity of treatment effect may arise when stratifying on an intermediate variable influenced by both treatment and baseline risk factors. Accordingly, it should be interpreted as a pedagogical example of a well-described causal structure rather than as empirical evidence specific to DAPA-CKD. This mechanism is well established in causal inference theory, and the general concern follows from causal structure rather than from any single parameterization. Furthermore, early eGFR decline is commonly modelled using fixed categorical thresholds. Yet the renal trajectory under sodium-glucose cotransporter 2 (SGLT2) inhibition comprises at least two biologically distinct phases: an acute haemodynamic shift followed by chronic slope modification [2]. Collapsing this dynamic process into static categories results in loss of trajectory information and may obscure heterogeneity in treatment response.

Joint longitudinal–survival modelling provides a coherent framework to address these concerns. Shared random-effects joint models enable simultaneous estimation of individual eGFR trajectories and time-to-event risk while accounting for measurement error and time-dependent associations [4]. This approach allows separation of acute and chronic slopes within a unified structure and permits more robust evaluation of whether the early haemodynamic response independently modifies subsequent renal risk. However, implementation of such models requires access to individual participant-level longitudinal and time-to-event data, which may not be readily available outside the original trial investigators. In addition, model specification and computational complexity may limit routine application in standard *post hoc* analyses. These limitations should be explicitly acknowledged when proposing this approach.

From a clinical perspective, these issues are not merely methodological. If subgroup analyses based on early eGFR decline are interpreted causally, clinicians may overinterpret stratum-specific estimates as evidence that the magnitude of the initial dip identifies patients with differential benefit from dapagliflozin, even when no true effect modification is specified in the underlying causal structure. This could inadvertently influence treatment continuation decisions in routine practice. Clear alignment between causal structure and analytical strategy is therefore essential to avoid misleading interpretations of treatment heterogeneity. Early eGFR decline should therefore be interpreted primarily as a treatment-induced haemodynamic response rather than as a reliable causal marker for withholding or discontinuing dapagliflozin, unless accompanied by clinical safety concerns.
